# A novel detection method based on MIRA-CRISPR/Cas13a-LFD targeting the repeated DNA sequence of *Trichomonas vaginalis*

**DOI:** 10.1186/s13071-023-06106-3

**Published:** 2024-01-08

**Authors:** Zhenke Yang, Jinghui Wang, Yiming Qi, Yiping Shi, Fakun Li, Weijuan Wang, Xiaowei Tian, Xuefang Mei, Zhenchao Zhang, Shuai Wang

**Affiliations:** 1 Xinxiang Key Laboratory of Pathogenic Biology, Department of Pathogenic Biology, School of Basic Medical Sciences, Xinxiang Medical University, Xinxiang, China; 2 Third Affiliated Hospital of Xinxiang Medical University, Xinxiang, China

**Keywords:** *Trichomonas vaginalis*, CRISPR/Cas13a, Diagnosis, Visualized, Lateral flow device

## Abstract

**Background:**

*Trichomonas vaginalis* is a protozoan parasite, widely recognized as the most prevalent non-viral sexually transmitted infection (STI) globally. This infection is linked to various complications, including pelvic inflammatory disease, adverse pregnancy outcomes, and an increased risk of acquiring HIV. Current molecular detection methods for *T. vaginalis* are often costly and technically challenging.

**Methods:**

We developed a novel detection method for *T. vaginalis* using a multi-enzyme isothermal rapid amplification–clustered regularly interspaced short palindromic repeats (MIRA-CRISPR)/Cas13a-lateral flow device (LFD). This assay targets the repeated DNA sequence (GenBank: L23861.1) of *T. vaginalis* and is performed at a constant temperature of 37 °C for approximately 1 hour.

**Results:**

The detection limit of genomic DNA (gDNA) using our protocol was 1 × 10^–4^ ng/μl. Specificity was confirmed by the absence of cross-reaction with gDNA from various other microorganisms such as *Staphylococcus aureus*, *Lactobacillus taiwanensis*, *Escherichia coli*, *Monilia albicans*, *Giardia lamblia*, or *Toxoplasma gondii*. Among 30 clinical samples tested, the positive rates of *T. vaginalis* detection were 33.33% (10/30) by wet mount microscopy, 40% (12/30) by nested polymerase chain reaction (PCR), 40% (12/30) by MIRA-CRISPR/Cas13a-LFD, and 40% (12/30) by the culture method. Compared with the culture method, the gold standard for diagnosing trichomoniasis, wet mount microscopy showed a sensitivity of 83.3% and moderate diagnostic agreement (kappa value = 0.87). Both nested PCR and MIRA-CRISPR/Cas13a-LFD exhibited 100% sensitivity and excellent diagnostic agreement (kappa value = 1).

**Conclusions:**

The MIRA-CRISPR/Cas13a-LFD method is a convenient, rapid, stable, and accurate diagnostic tool for detecting *T. vaginalis*. This method has the potential to enhance the diagnosis and management of vaginitis, offering a significant improvement over existing diagnostic techniques.

**Graphical Abstract:**

**Figure:**
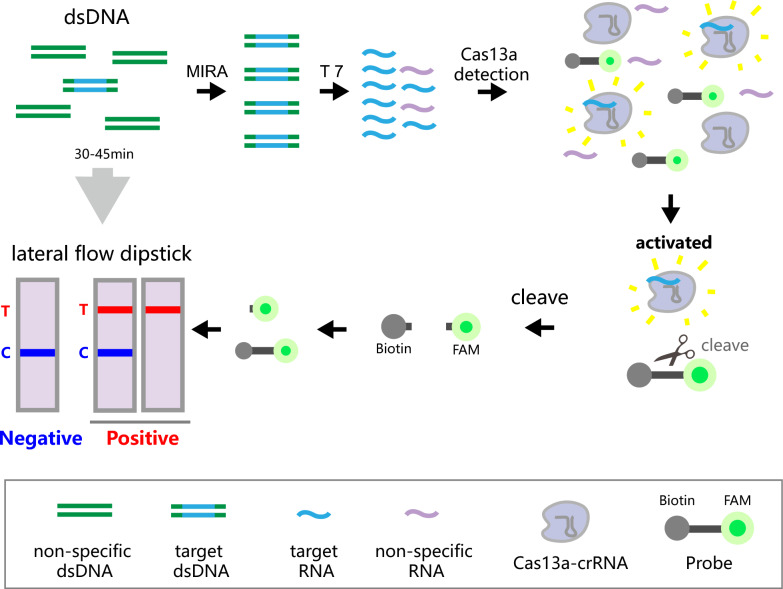

**Supplementary Information:**

The online version contains supplementary material available at 10.1186/s13071-023-06106-3.

## Background

*Trichomonas vaginalis* is a flagellated, protozoan parasite, recognized as the most common non-viral sexually transmitted infection (STI) globally, with an estimated 276 million new cases reported annually; the global prevalence of trichomoniasis has been estimated at 5.3% in females and 0.6% in males, with a growing trend [1, 2]. Despite its ubiquity, it is often underdiagnosed and undertreated due to its asymptomatic nature in a significant proportion of cases [3]. However, when symptoms do occur, they can lead to serious health complications, including pelvic inflammatory disease, adverse pregnancy outcomes, and increased susceptibility to HIV [4]. Moreover, pregnant women afflicted with this infection may be at higher risk of premature birth and lower birth weight [5]. Hence, prompt and accurate identification of *T. vaginalis* is of utmost significance from a clinical standpoint.

Currently, diagnostic approaches for *T. vaginalis* infection predominantly involve wet mount microscopy, culture techniques, antigen detection, loop-mediated isothermal amplification (LAMP), and nucleic acid amplification tests (NAATs) [6–9]. However, these techniques have considerable limitations. Wet mount microscopy, while quick and inexpensive, suffers from low sensitivity, and the culture method, despite its high sensitivity, is time-consuming and labor-intensive [10]. Antigen detection lacks sensitivity, particularly in males, while NAATs, though highly sensitive and specific, require sophisticated laboratory equipment and skilled personnel, and are relatively costly [11]. These constraints hinder the broad implementation of these diagnostic methods, especially in resource-limited settings.

Multi-enzyme isothermal rapid amplification (MIRA) is an isothermal amplification method that combines multiple enzymes to achieve rapid and efficient amplification of target nucleic acids. MIRA has been widely applied in diagnostic settings due to its advantages such as simplicity, rapidity, and sensitivity. It has been used for the detection of various pathogens, including bacteria and viruses [12, 13]. However, MIRA also has some limitations. One major drawback is the potential for non-specific amplification, leading to false-positive results. This can occur due to primer–dimer formation, off-target binding, or the presence of non-specific DNA templates.

The clustered regularly interspaced short palindromic repeats (CRISPR) and CRISPR-associated (Cas) proteins were initially discovered as part of the bacterial immune system, enabling the recognition and cleavage of foreign genetic material [14]. The Cas13a protein, in particular, has been repurposed for diagnostic applications due to its ability to cleave RNA sequences with high precision. The system operates by programming the Cas13a protein with a guide RNA (gRNA) that targets a specific RNA sequence of interest. Upon successful recognition of the target RNA, Cas13a is activated and exhibits collateral cleavage activity, indiscriminately targeting nearby RNA molecules. This collateral cleavage activity can be harnessed as a signal for the detection of the pathogenic RNA, providing a sensitive and specific diagnostic tool [15]. Given the outstanding specificity of the CRISPR/Cas13 system in RNA recognition, its combination with isothermal amplification technology (IAT) proves to be a potent solution for addressing limitations related to specificity in pathogen detection. Numerous studies have showcased the efficacy of CRISPR/Cas13a in detecting various pathogens, including severe acute respiratory syndrome coronavirus 2 (SARS-CoV-2) [16], Ebola virus [17], and *Schistosoma japonicum* [18].

In this study, we present a novel diagnostic approach for *T. vaginalis*, combining MIRA, CRISPR/Cas13a, and lateral flow device (LFD) targeting. Isothermal amplification simplifies the nucleic acid amplification process by eliminating the need for thermal cycling, thus reducing the complexity and cost [19]. CRISPR/Cas13a is known for its high sensitivity and specificity in RNA targeting and cleavage, allowing for accurate identification of the pathogen [20]. Lastly, LFDs offer a user-friendly, rapid, and visual detection method [21]. We believe that this integrated diagnostic approach could significantly improve the detection of *T. vaginalis*, offering a solution that is not only rapid and highly accurate but also suitable for point-of-care testing, particularly in resource-constrained environments.

## Methods

### Plasmid construction and primers

The *T. vaginalis* repeated element (GenBank: L23861.1) is composed of more than 100 genes of similar sequences in the genome, based on analysis using the Basic Local Alignment Search Tool (BLAST) in the National Center for Biotechnology Information (NCBI) database (data not shown), and therefore is considered the potential optimal target for *T. vaginalis* diagnosis. In this study, we amplified the sequence of a 1019-base-pair fragment from *T. vaginalis* genomic DNA (gDNA) using the primers below (forward: 5′-cccgaattcCACACCCCAAACTTGCAATGACACT-3′, reverse: 5′-cccaagcttGGATGGGAAATTAAGGGTAATTTTCCA-3′), and cloned the fragment into vector pUC19 (Vazyme, China) with HindIII and EcoRI to construct pUC19-L23861.1 positive recombinant plasmid. The plasmid was used as standard nucleic acids to quantify the sequences with a NanoDrop 2000 spectrophotometer (Thermo Fisher Scientific, USA). Then, the recombinant plasmids were prepared with a dilution range of 1 ng/μl to 1 × 10^−9^ ng/μl as a standard control and stored at −20 °C for further use. All the primers used in this study are listed in Table 1.

**Table 1:** Primers and probes used in this study

Primers	Sequences (5′–3′)	Description
TV-F1	TCCATTAAGAAACCTCAAGAGATGACAAGAG	
TV-F2	ATTGCTTATGAATTCGACATGGGATATTCC	
TV-F3	ACCCCAAACTTGCAATGACACTCAAATTGC	
TV-R1	TAATACGACTCACTATAGGGACTGAGGGTAAGACCAATGTTCGACAATAG	
TV-R2	TAATACGACTCACTATAGGGTTGTTGTAGTTGTCAAGGACTGCCTTTGCG	Candidates for MIRA primers screened in this study TV-F5/TV-R5 pair was used for follow-up experimental tests (this paper)
TV-R3	TAATACGACTCACTATAGGGACCTTCACTTTGGATTTGACTTCGGAGAAG	
TV-F4	TCGACATGGGATATTCCATTAAGAAACCTC	
TV-F5	ACTTGCAATGACACTCAAATTGCTTATGAA	
TV-F6	ACACCCCAAACTTGCAATGACACTCAAATTGCTTA	
TV-F7	TGCAATGACACTCAAATTGCTTATGAATTCG	
TV-R4	TAATACGACTCACTATAGGGTTGCGAACTGAGGGTAAGACCAATGTTCGACAATAGGT	
TV-R5	TAATACGACTCACTATAGGGTACTAAGCCACTTTGACCTTGAGTGAATTCGTACTTC	
TV-R6	TAATACGACTCACTATAGGGAATGTTCGACAATAGGTTTTAATGTGGTTACTAAGCCAC	
TV-R7	TAATACGACTCACTATAGGGAGACCAATGTTCGACAATAG	
TV-crRNA	GAUUUAGACUACCCCAAAAACGAAGGGGACUAAAACUUAUGAGAAGGUCGACAACUUUGUUGAA	crRNA (this paper)
13U-FB-reporter	FAM-UUUUUUUUUUUUU-Bio	Reporter
6U-FB-reporter	FAM-UUUUUU-Bio	
TV-8S	TCTGGAATGGCTGAAGAAGACG	Nested PCR primers [26] (actin gene)
TV-9R	CAGGGTACATCGTATTGGTC	
TV-10S	CAGACACTCGTTATCG	
TV-11R	CGGTGAACGATGGATG	

*TV* *Trichomonas vaginalis*, *FB* FAM (carboxyfluorescein) and biotin

### Parasites

The *T. vaginalis* strain used in this study was isolated from the vaginal secretions of female patients diagnosed with trichomoniasis. The strain was identified as actin genotype E, which is the predominant genotype in Xinxiang City, Henan Province, China [22]. Following the protocol outlined in our previous study [23], *T. vaginalis* was cultured in complete TYM (trypticase–yeast extract–maltose) medium at 37 °C in a humidified chamber with 5% CO_2_. The parasites were harvested from the stationary phase by centrifugation, and DNA extraction was performed using 5 × 10^6^ trophozoites for subsequent analyses.

### DNA extraction and DNA samples

Trophozoites of *T. vaginalis* were washed three times with phosphate-buffered saline (PBS) (pH 7.4) and then isolated by centrifugation (1500×*g*, 5 min). The DNA extraction from *T. vaginalis* trophozoites was performed using commercial kits (#D3396, Omega Bio-tek, USA) following the manufacturer's instructions. The DNA samples used in this study, including *Staphylococcus aureus*, *Lactobacillus taiwanensis*, *Escherichia coli*, *Monilia albicans*, *Giardia lamblia*, and *Toxoplasma gondii*, were obtained from the Department of Pathogenic Biology, Xinxiang Medical University. All DNA samples were stored at −20 °C before use.

### MIRA-CRISPR/Cas13a-LFD assay

The MIRA reactions were conducted utilizing the MIRA basic kit (#WLB8201KIT, Amp-Future Biotech Co., China). Briefly, each assay comprised a final reaction volume of 50 µl, consisting of buffer A (29.4 µl), DNA template (10 µl), double-distilled water (ddH_2_O) (4.1 µl), forward primer (2 µl), reverse primer (2 µl), and 2.5 µl of buffer B. Incubation of the mixture was carried out at 37 °C for 30 min. Positive control was established using positive plasmid pUC19-L23861.1, while ddH_2_O served as the blank control for each batch of MIRA reaction mixture.

Following the MIRA reaction, the resulting MIRA products served as the input for the CRISPR/Cas13a-LFD assay in a total reaction volume of 10 μl. Specifically, 1 μl of MIRA products was combined with the CRISPR reaction mixture, including 1 μl of CRISPR RNA (crRNA) probe (1 μM) (see Table 1), 1 μl of LwCas13a nuclease (5 μM) (#EDE0001, Editgene, China), 1 μl of lateral flow reporter molecule (1 μM) (see in Table 1), RNase inhibitor (1 μl, 40U/μl) (New England Biolabs, USA), ATP (1 μl, 100 mM), GTP (1 μl, 100 mM), UTP (1 μl, 100 mM), CTP (1 μl, 100 mM) (#B600059-0001 NTP Set Solution, Sangon Biotech, China), and T7 polymerase (1 μl, 50U/μl) (#DD4101-02, Vazyme, China). After thorough mixing and incubation at 37 °C for 30 min, 20 μl of ddH_2_O was added to the 10 μl reaction, followed by another round of thorough mixing. The mixture was then inserted into the LFD (#JY0301, Tiosbio, China) and incubated for 3 min while recording the results until the positive control line became visible. No components of this assay were constructed in our laboratory, and all molecular tools were commercially available.

### Specificity and sensitivity tests

To assess the analytical specificity of the MIRA-CRISPR/Cas13a-LFD assay developed in this study, nucleic acids from various microorganisms were employed as templates for the MIRA reaction. Specifically, the nucleic acids of *S. aureus*, *L. taiwanensis*, *E. coli*, *M. albicans*, *G. lamblia*, and *T. gondii* were included. For sensitivity analysis of the MIRA-CRISPR/Cas13a-LFD assay, 10-fold serial dilutions of recombinant plasmids were prepared, ranging from 1 × 10^–7^ ng/μl to 1 × 10^–9^ ng/μl, as well as genomic DNA (gDNA) ranging from 1 × 10^–2^ ng/μl to 1 × 10^–4^ ng/μl. In some experiments, duplicates were performed using recombinant plasmid as the positive control, while ddH_2_O served as the negative control.

### Clinical samples used in this study

Vaginal secretions were obtained from 30 women presenting clinical symptoms of trichomoniasis at the Third Affiliated Hospital of Xinxiang Medical University, following collection by medical staff using swabs; the volume of each sample was approximately 1 ml. To establish *T. vaginalis* cultures, clinical samples (50 μl) were inoculated into fresh TYM medium and incubated at 37 °C in a 5% CO_2_ environment for 72 h. For DNA extraction, 800 μl of the sample was processed using the E.Z.N.A.^®^ Tissue DNA kit (#D3396-02, Omega Bio-tek, USA). Following DNA extraction, 100 ng of DNA was utilized for each reaction in nested PCR, and 100 pg of DNA was employed for MIRA-CRISPR/Cas13a-LFD detection.

### Wet mount microscopy

The wet mount microscopy followed a procedure described in a previous study [24]. Briefly, a small amount of the collected vaginal discharge was smeared on a clean glass microscope slide, and a drop of saline (0.9% NaCl solution) was added. Next, a cover slip was carefully placed over the sample on the slide to avoid any air bubbles that might distort the view. The wet mount was then immediately examined under the microscope, starting with the low-power objective (×10) to locate the field and then switching to the high-power objective (×40) to look for *T. vaginalis*, which are pear-shaped protozoa with four anterior flagella and an undulating membrane. If motile *T. vaginalis* were seen, the test was positive. If no *T. vaginalis* were seen, the test was negative.

### Nested PCR

The actin gene (GenBank: AF237734) was chosen as the target gene for nested PCR amplification [25]. Based on primer sequences reported in the literature [26], the nested PCR was performed in two rounds. In the first round, the PCR amplification system contained 2× Taq Plus Master Mix (Dye Plus) Enzyme (12.5 μl), 1 μl each of upstream and downstream primers, 1 μl of template, and ddH_2_O to reach a final volume of 25 μl. The first round of PCR included an initial denaturation step at 95 °C for 3 min, followed by 35 cycles of denaturation at 95 °C for 15 s, annealing at 55 °C for 15 s, and extension at 72 °C for 1 min. A final extension was performed at 72 °C for 5 min. For the second round, 2 μl of the product from the first round was used as the template. The reaction mixture for the second round contained 2× Dye Plus Enzyme (25 μl), 2 μl each of upstream and downstream primers, and ddH_2_O to reach a final volume of 50 μl. The second round of PCR followed a similar protocol with an initial denaturation step at 95 °C for 3 min, followed by 35 cycles of denaturation at 95 °C for 15 s, annealing at 50 °C for 15 s, and extension at 72 °C for 1 min. A final extension was performed at 72 °C for 5 min. The amplified products were then analyzed using 1.0% agarose gel electrophoresis.

### Statistical analysis

Statistical analysis was performed to evaluate the diagnostic performance of wet mount microscopy, culture, nested PCR, and MIRA-CRISPR/Cas13a-LFD assays in this study. Key parameters including sensitivity and specificity were calculated. The diagnostic standard employed for comparison was the culture method. The level of agreement between each diagnostic method and culture was assessed using the kappa test in SPSS version 27.0. The agreement between the diagnostic methods and culture was categorized based on kappa values, as follows: excellent (1.00–0.81), substantial (0.80–0.61), moderate (0.60–0.41), weak (0.40–0.21), and negligible (0.20–0) [27]. This analysis enabled the determination of the level of agreement between each method and the gold standard culture, providing insight into the diagnostic performance of the different assays.

## Results

### Rapid detection strategy for *T. vaginalis*

**Fig. 1:**
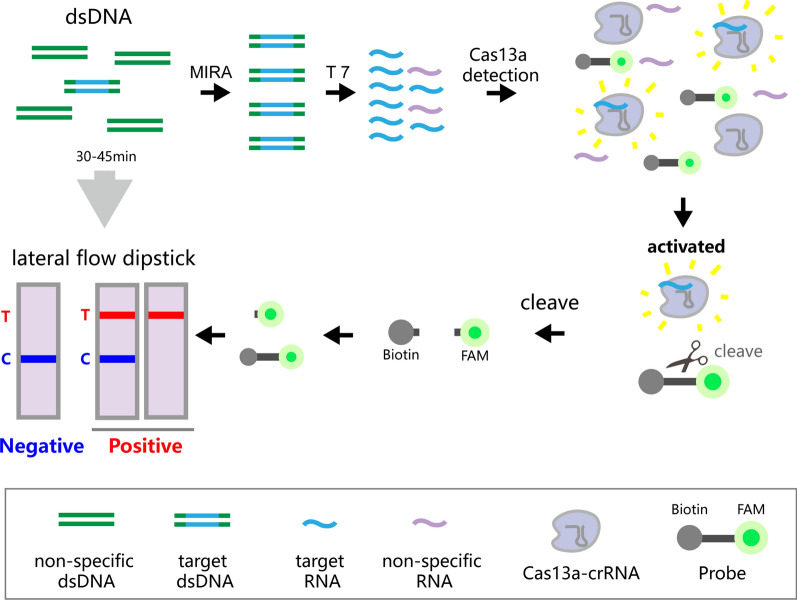
Schematic for *T. vaginalis* detection with the MIRA-CRISPR/Cas13a-LFD assay

In the pursuit of rapid and visualized diagnostic strategies for *T. vaginalis*, we have meticulously designed a detailed process as illustrated in Fig. 1. The diagnostic protocol commences with the amplification of the target sequence via MIRA. This amplified product is subsequently transcribed into single-stranded RNA (ssRNA) using the T7 RNA polymerase promoter. A crRNA was meticulously selected as a probe to target the specific ssRNA. Upon recognition of the target ssRNA, the non-specific ribonuclease endonuclease activity of the CRISPR/Cas13a complex is activated, resulting in cleavage of the probe substrate. In the absence of trans-cleavage, the carboxyfluorescein (FAM)/biotin (FB) reporter remains undamaged, allowing its biotinylated end to be captured by streptavidin at the control line. This then permits the anti-FAM antibody-conjugated gold nanoparticles to bind to the exposed FAM moiety, which results in a color deposit at the control line. The appearance of only the control band (blue band) is indicative of negative test results, implying unsuccessful amplification of the target gene fragment within the sample. Conversely, when the FB reporter is subjected to trans-cleavage by Cas13a, biotin will occupy a section of the reporter molecules, replacing FAM. Consequently, fewer FAM ends will be visible at the control line, while a greater number of antibody-conjugated gold nanoparticles are likely to aggregate at the test line. If both the test line and control line, or solely the test line, are visible, the test is deemed positive. This would signify the successful detection of the *T. vaginalis* target gene within the samples.

### *Trichomonas vaginalis* repeat DNA sequence (L23861.1) as target for diagnosis

To maximize the amplification efficiency of our target gene and enhance diagnostic sensitivity, we selected the repetitive DNA sequence (L23861.1) of *T. vaginalis* as the target gene. Initially, we designed three sets of MIRA primers, namely TV-F1/F2/F3 and TV-R1/R2/R3 (Table 1).

**Fig. 2:**
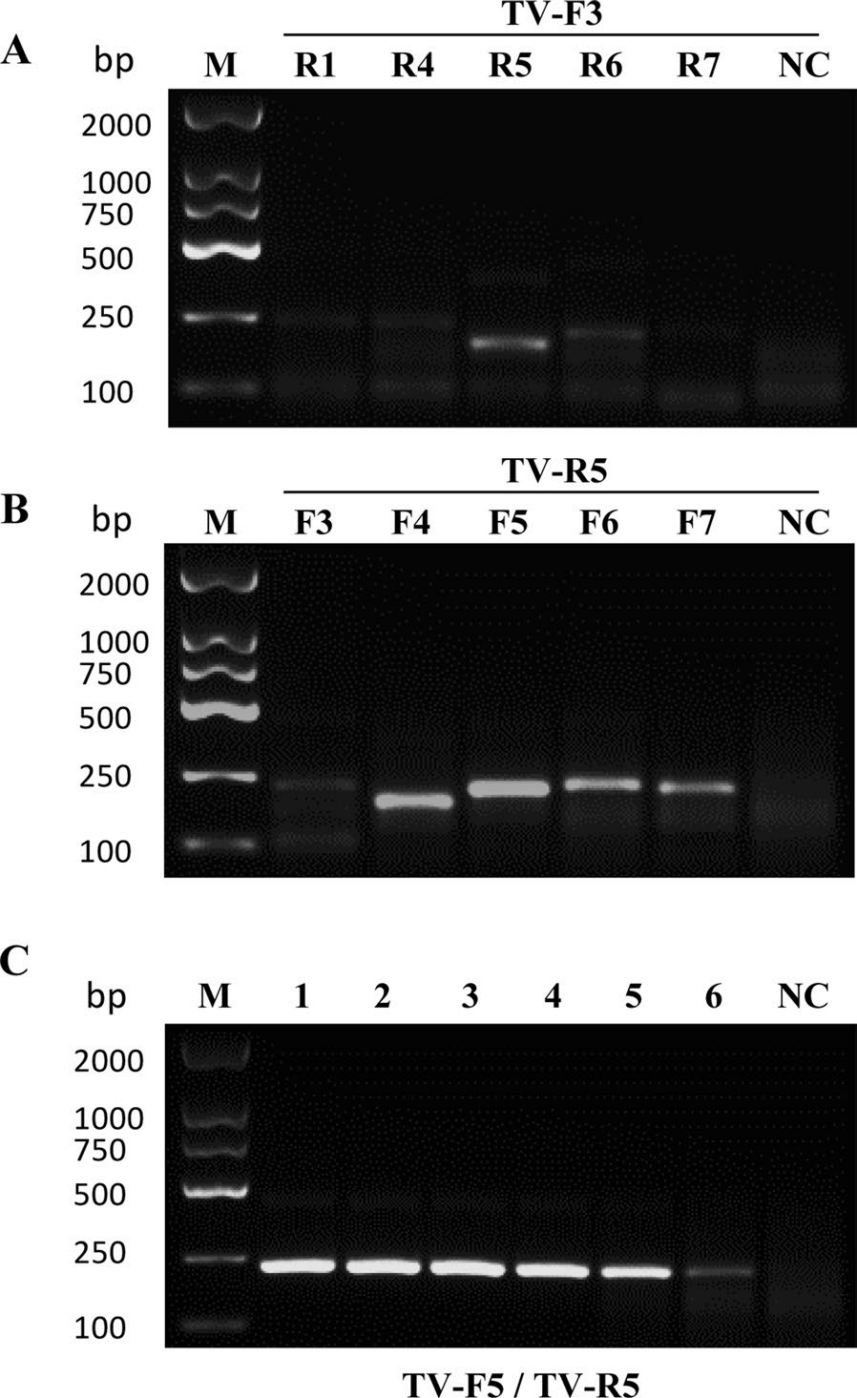
Primer selection for MIRA of *T. vaginalis* repetitive DNA sequence. **A** Screening of the optimal reverse primer using TV-F3 as the forward primer. **B** Screening of the optimal forward primer using TV-R5 as the reverse primer. **C** Sensitivity testing of the TV-F5/TV-R5 primer pair using positive plasmid template concentrations as follows. 1: 1 × 10^–2^ ng/μl;2: 1 × 10^–3^ ng/μl; 3: 1 × 10^–4^ ng/μl; 4: 1 × 10^–5^ ng/μl; 5: 1 × 10^–6^ ng/μl; 6: 1 × 10^–7^ ng/μl. *NC* negative control

Using positive plasmid as the template and TV-R1 as the reverse primer, we assessed the efficacy of MIRA forward primers. Our results showed better amplification efficiency for TV-F3 (Additional file 1: Figure S1A). Subsequently, when using TV-F3 as the forward primer, TV-R1 exhibited the highest amplification efficiency (Additional file 1: Figure S1B). Performance testing was then carried out on the primer pair TV-F3 and TV-R1 using plasmid templates at varying concentrations, including 10 pg/μl, 1 pg/μl, 100 fg/μl, 10 fg/μl, 1 fg/μl, and 0.1 fg/μl. Notably, sensitivity as low as 10 fg/μl was observed (Additional file 1: Figure S1C). These findings prompted us to redesign four primer pairs and subsequently subject them to a screening process. Remarkably, TV-F5 and TV-R5 exhibited excellent specificity and demonstrated sensitivity of 0.1 fg/μl when utilizing the template plasmid (Fig. 2A–C).

### MIRA time and temperature optimization

**Fig. 3:**
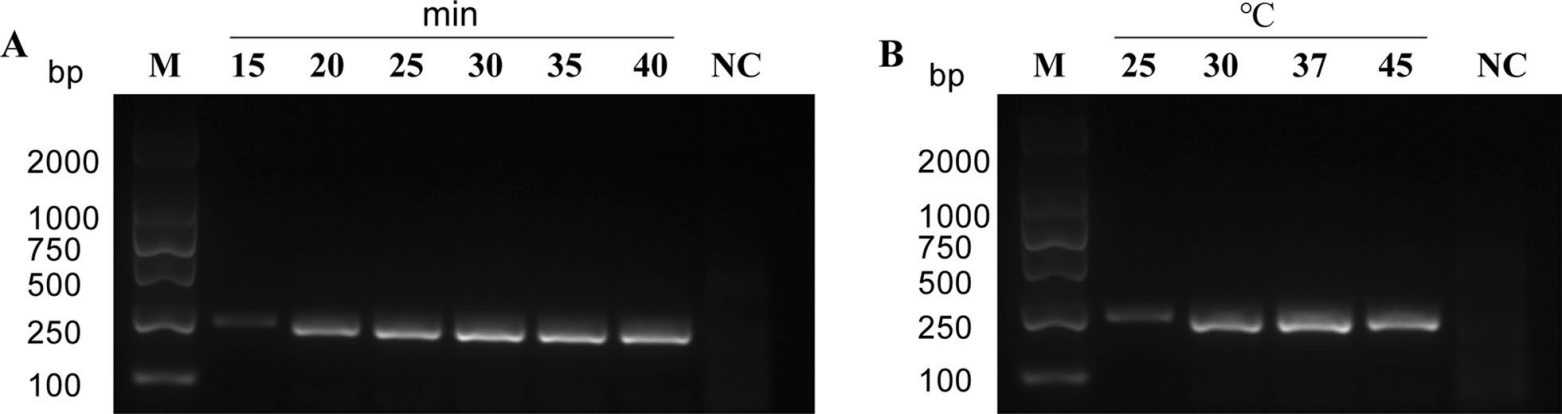
MIRA time and temperature optimization. **A** Optimal reaction time for MIRA assay. Saturation amplification was achieved within 30 min. **B** Temperature optimization for MIRA from 25 °C to 45 °C; the highest amplification efficiency was observed at 37 °C. *NC* negative control

To enhance the performance of isothermal amplification in vitro, we conducted optimization of amplification time and temperature by creating gradients of both variables. Our findings indicate that the most favorable amplification efficiency of MIRA was achieved after 30 min at a temperature of 37 °C (Fig. 3A and B).

### MIRA-CRISPR/Cas13a-LFD system for *T. vaginalis* detection

**Fig. 4:**
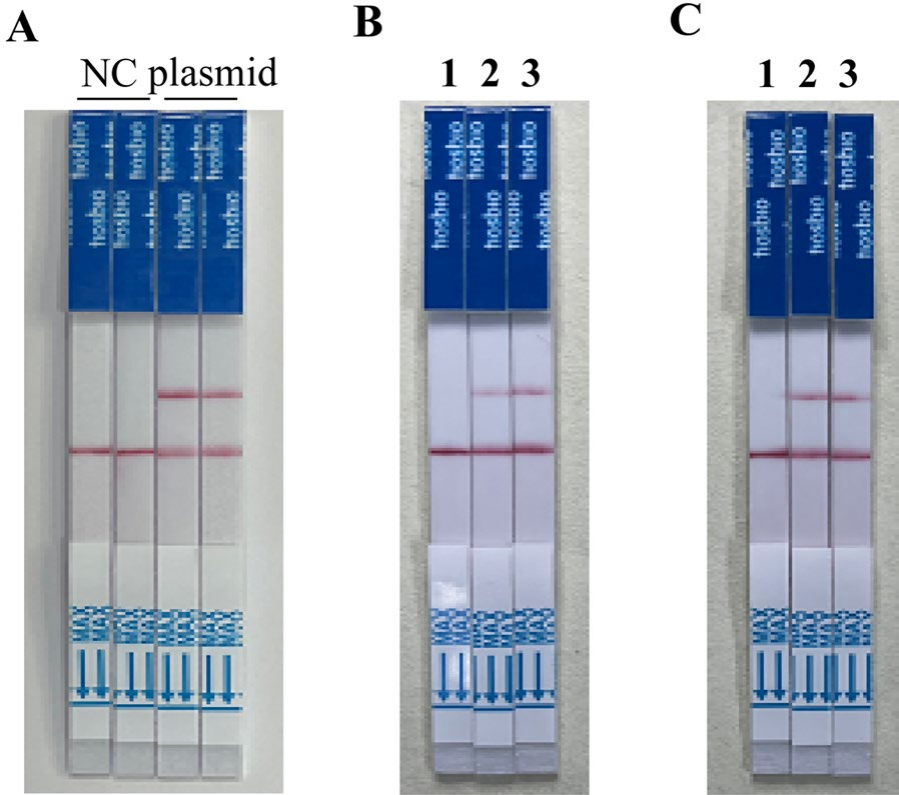
MIRA-CRISPR/Cas13a-LFD system test for *T. vaginalis* detection. **A** MIRA-CRISPR/Cas13a-LFD system test, NC: negative control using ddH_2_O as template. Plasmid: testing group using 1 × 10^–6^ ng/μl positive plasmid as the template. **B**, **C** MIRA-CRISPR/Cas13a-LFD assay FAM-UUUUUUUUUUUUU-biotin reporter (13U-FB-reporter) and FAM-UUUUUU-biotin reporter (6U-FB-reporter) test. 1. Negative control using ddH_2_O as template. 2. 1 × 10^–6^ ng/μl positive plasmid as the template. 3. 1 × 10^–3^ ng/μl positive plasmid as the template

If an excessive number of probes are present in the system, it may result in the saturation of the C line of LFD by uncut probes, and this saturation can lead to their leakage through to the T line, causing false-positive results [28]. Hence, we performed preliminary tests to assess the impact of probe concentrations on the reliability of the results. Our investigation revealed that when employing CRISPR probes at concentrations of 500, 200, 100, and 50 nM for direct lateral flow detection, devices in the 500 nM concentration group exhibited a notable occurrence of false-positive bands, while the other concentration groups did not display such issues (Additional file 2: Fig S2). Thus, to maintain the reliability of our results, the probe concentration was kept below 200 nM. The pUC19-L23861.1 plasmid (10 fg/μl) was used as template and amplified using MIRA with TV-F5 and TV-R5 primers, employing conditions of 37 °C for 30 min. Based on the crRNA and the FAM-13U-biotin reporter (13U-FB reporter) [29] (Table 1), we designed and optimized a reaction system (see Methods section) for CRISPR/Cas13a-LFD detection. The results showed that compared with the control group, the plasmid group exhibited strong and distinct bands (Fig. 4A). To further optimize the system, we designed a new probe, FAM-6U-biotin reporter (6U-FB reporter), reducing the cleaved nucleic acid chain from 13 to 6 uracil. Interestingly, when comparing the band patterns of samples with different plasmid concentrations (1 fg/μl and 1 pg/μl) under the conditions of 6U-FB reporter and 13U-FB reporter, no significant difference was observed, indicating that the probes could be used interchangeably for further experiments (Fig. 4B and C).

### Specificity test

**Fig. 5:**
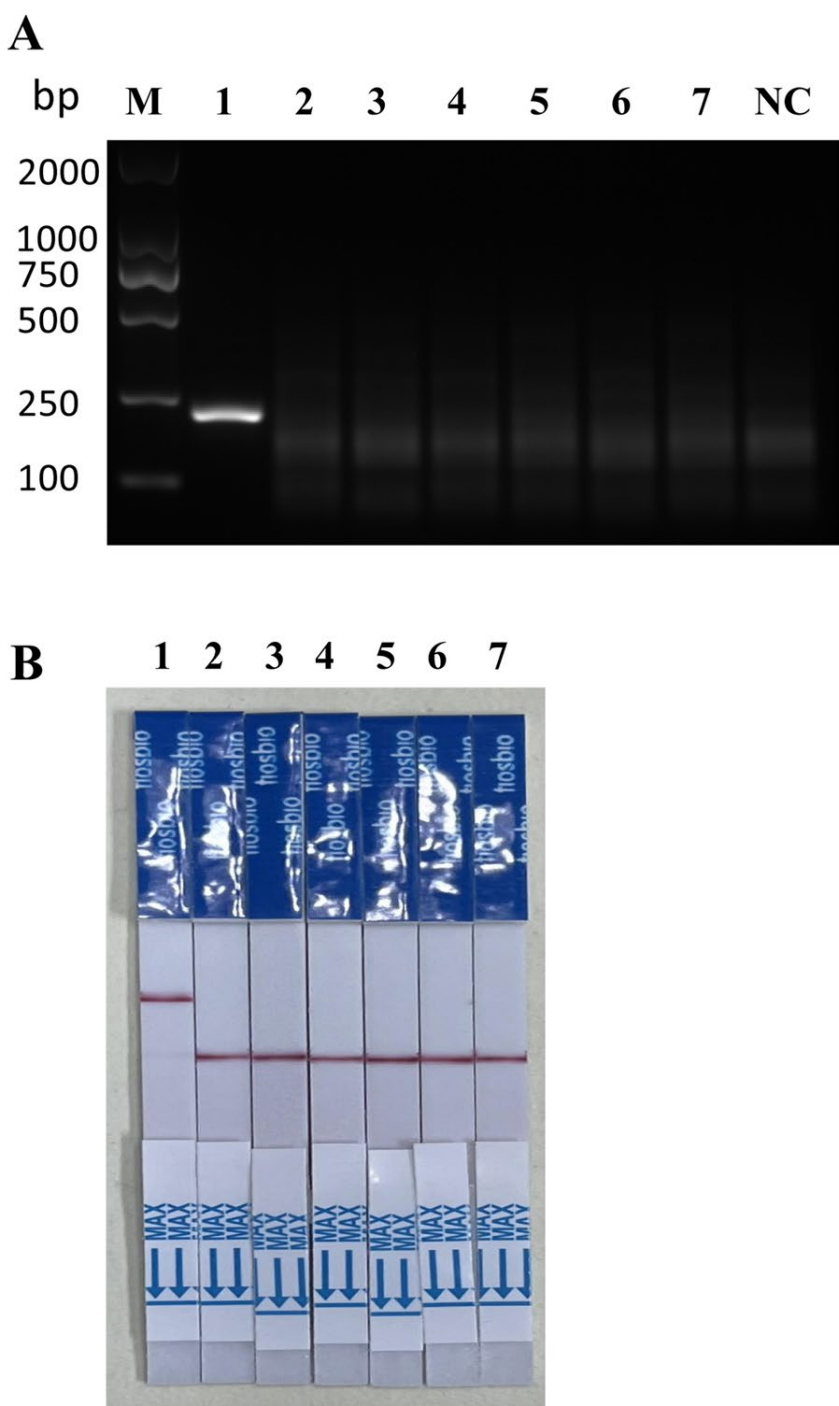
MIRA-CRISPR/Cas13a-LFD specificity test. **A** Specificity analysis of MIRA assay. **B** Specificity analysis of MIRA-CRISPR/Cas13a-LFD assay. 1. *Trichomonas vaginalis*; 2. *Staphylococcus aureus*; 3. *Lactobacillus taiwanensis*; 4. *Escherichia coli*; 5. *Monilia albicans*; 6. *Giardia lamblia*; 7. *Toxoplasma gondii*; NC: negative control using ddH_2_O as template

Using the optimized experimental parameters, we assessed the diagnostic specificity of the method employed in this study. To this end, we included gDNA samples from six common pathogens in humans, namely *S. aureus*, *L. taiwanensis*, *E. coli*, *M. albicans*, *G. lamblia*, and *T. gondii*, as controls for MIRA isothermal amplification. It is worth mentioning that despite the presence of significant non-specific bands observed during the MIRA isothermal amplification process (Fig. 5A), the CRISPR/Cas13a complex exhibited no recognition of off-target gene sequences. Consequently, the probe remained intact without undergoing cleavage, leading to negative LFD results (Fig. 5B). This finding underscores the robustness of our approach in identifying *T. vaginalis.*

### Sensitivity test

**Fig. 6:**
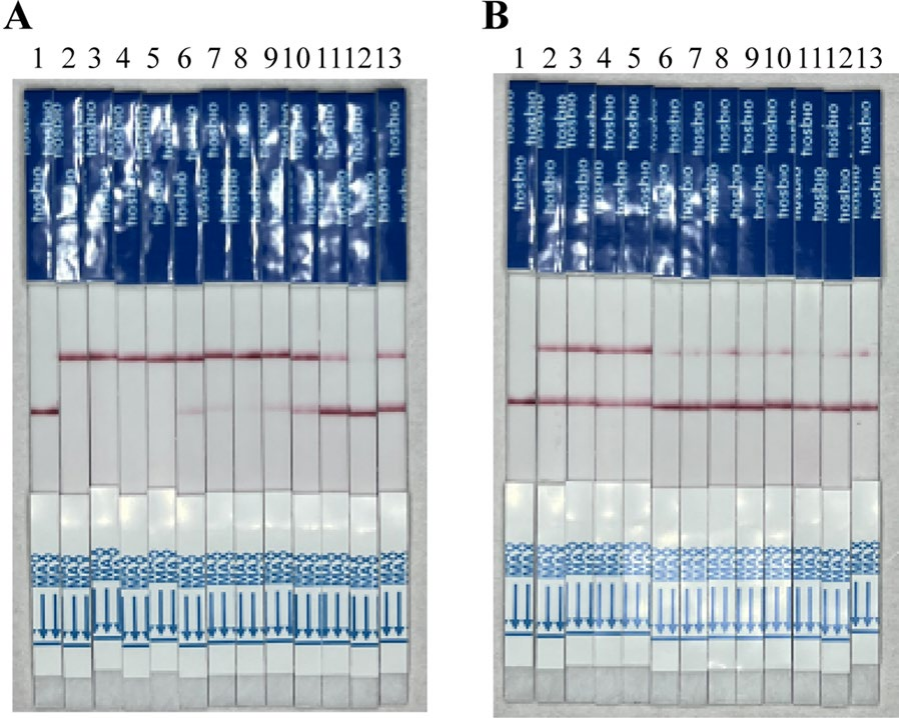
MIRA-CRISPR/Cas13a-LFD sensitivity test. Different concentrations of positive plasmid DNA (**A**) and *T. vaginalis* gDNA (**B**) were used for the MIRA-CRISPR/Cas13a-LFD assay. **A** 1: Negative control; 2–5: 0.1 fg/μl; 6–9: 0.01 fg/μl; 10–13: 0.001 fg/μl. **B** 1: Negative control; 2–5: 10 pg/μl; 6–9: 1 pg/μl; 10–13: 100 fg/μl

The sensitivity of this diagnosis system was evaluated using serial dilutions of positive plasmid and *T. vaginalis* genomic DNA. We found that our MIRA-CRISPR/Cas13a-LFD system could reliably detect as little as 0.01 fg/μl of positive plasmid and 1 pg/μl of *T. vaginalis* gDNA. When the plasmid concentration was 0.001 fg/μl and the genomic DNA concentration was 100 fg/μl, the test's positivity rate also reached 75% (3/4) (Fig. 6A and B).

### Assessment of the MIRA–Cas13a–LFD assay on clinical samples

**Fig. 7:**
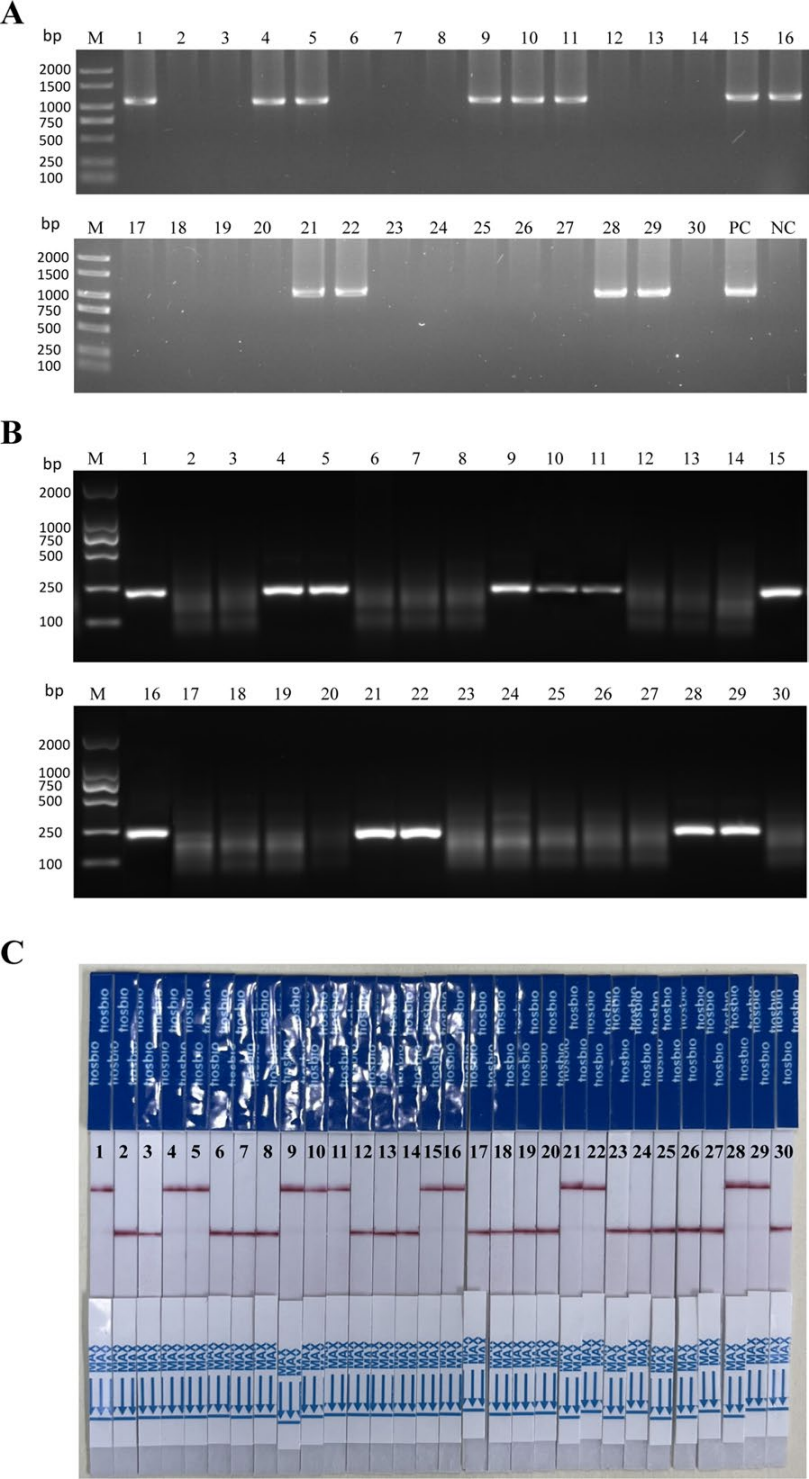
*Trichomonas vaginalis* detected in clinical samples via MIRA-CRISPR/Cas13a-LFD and other methods **A** Nested PCR diagnosis of 30 samples presenting symptoms of trichomoniasis. *PC* positive control, *NC* negative control. **B** MIRA direct diagnosis of the 30 clinical samples. **C** MIRA-CRISPR/Cas13a-LFD assay platform results of 30 female samples were interpreted using an LFD sensor

Vaginal discharge samples were collected from 30 patients clinically diagnosed with vaginitis. These samples were concurrently tested using four different diagnostic methods: conventional culture method (gold standard), wet mount microscopy, nested PCR, and the newly developed MIRA-CRISPR/Cas13a-LFD technique presented in this study. Out of the 30 samples tested, the positive detection rates yielded by the culture method, wet mount microscopy, nested PCR (Fig. 7A), and MIRA-CRISPR/Cas13a-LFD (Fig. 7B and C) were 40% (12/30), 33.3% (10/30), 40% (12/30), and 40% (12/30), respectively. When compared with the gold standard culture method, the sensitivity of the wet mount microscopy was only 83.3%. In contrast, both nested PCR and the MIRA-CRISPR/Cas13a-LFD methods demonstrated 100% efficiency, aligning perfectly with the results of the culture method.

To evaluate the level of agreement between these diagnostic methods and the gold standard, a kappa statistical analysis was performed. The wet mount microscopy method only reached a kappa value of 0.87, suggesting that this method often results in missed detection. On the other hand, both nested PCR and the MIRA-CRISPR/Cas13a-LFD methods achieved a kappa value of 1, showing an excellent level of agreement with the gold standard culture method (Table 2). These results suggest that the MIRA-CRISPR/Cas13a-LFD method is not only convenient and quick but also stable and accurate and would potentially enhance the diagnosis and management of vaginitis.

**Table 2:** Comparison of methods for detection of *T. vaginalis* by testing clinical samples (*n* = *30*)

Method	No. positive	Positive rate (%)	Sensitivity (%)	Kappa
Wet mount microscopy	10	33.3	83.3	0.87
Nested PCR	12	40	100	1
MIRA-CRISPR/Cas13a-LFD	12	40	100	1
Culture	12	40	100	1

The sensitivity of the tests was determined using the culture method as the standard

## Discussion

*Trichomonas vaginalis* is a flagellated protozoan parasite that is primarily known to cause trichomoniasis, one of the most common non-viral STIs globally [30]. The clinical manifestations of the infection range from asymptomatic to severe inflammatory disease. Given the widespread prevalence and significant health consequences of *T. vaginalis* infection, there is an urgent need for rapid, accurate, and easily interpretable diagnostic methods. Early and accurate detection of *T. vaginalis* not only can facilitate timely treatment and prevent adverse health outcomes, but can also aid in reducing transmission rates [31].

Nested PCR has long been established as a sensitive and reliable method for the detection of various pathogens, including *T. vaginalis* [32]. Our study confirmed that nested PCR maintains sensitivity and specificity on par with the gold standard in pathogen detection, effectively identifying *T. vaginalis*. However, the inherent sensitivity of nested PCR comes at the cost of complexity, time, and resource requirements. It involves multiple amplification steps, increasing the risk of contamination and necessitating meticulous laboratory conditions. Additionally, the method typically requires skilled personnel and advanced laboratory infrastructure, limiting its applicability in resource-limited settings and at the point of care [33]. IAT, although slightly inferior in specificity, represents a significant advancement from nested PCR. It simplifies the amplification of target genes in direct scenarios, eliminating the need for expensive equipment, and has been developed for diagnosing *T. vaginalis* [34]. The integration of IAT with LFD for visualizing detection results has been successfully reported in detecting viruses [35], bacteria [36], and parasites [37]. However, its application in *T. vaginalis* detection remains unreported, likely due to challenges in ensuring adequate specificity. Our study demonstrates a novel detection method for *T. vaginalis* by combining the benefits of MIRA with the CRISPR/Cas13a system and readout in LFD format. The strength of our diagnostic approach lies in its ability to leverage CRISPR/Cas13a's non-specific ribonuclease activity upon target recognition, which yields clear and concise diagnostic outcomes on a lateral flow dipstick.

In this study, the MIRA-CRISPR/Cas13a-LFD system showed remarkable efficiency and sensitivity in amplifying and detecting the *T. vaginalis* repeat DNA sequence (L23861.1), and the system's capacity to accurately detect as low as 10 fg/µl of the target gene with no false-positive results is noteworthy. One of the main findings of our study is the remarkable specificity of the MIRA-CRISPR/Cas13a-LFD system, even in the presence of non-specific bands observed during the MIRA isothermal amplification process. This implies that the CRISPR/Cas13a system could effectively avoid off-target gene sequences, thereby minimizing false positives, which is a significant challenge in diagnostic tests.

The differential detection capability of our proposed method is notable, distinguishing *T. vaginalis* from several other microorganisms including *L. taiwanensis*, *E. coli*, *G. lamblia*, *S. aureus*, and *M. albicans*. This is critical in clinical settings, where accurate diagnosis is paramount for successful treatment.

Further, in a clinical setting, our system consistently exhibited robust diagnostic results. The detection rate of our system, when compared with the conventional culture method (gold standard), wet mount microscopy, and nested PCR, proved to be highly satisfactory. In fact, the sensitivity of the MIRA-CRISPR/Cas13a-LFD method was equivalent to nested PCR and superior to wet mount microscopy.

Intriguingly, another recent research work adopted a comparable isothermal amplification technique integrated with CRISPR-Cas12a and a lateral flow strip for the detection of *T. vaginalis*. Although their methodology also sidesteps the requirement for a complex laboratory apparatus, the key difference lies in our choice of target gene, which may enhance our system's sensitivity, but due to differences in the method for calculating sensitivity, it remains to be conclusively proven which technique holds the upper hand [38].

Despite the promising outcomes, certain limitations associated with our approach need to be acknowledged. First, the system’s potential for mass production and global distribution needs to be validated through further studies. Given the need for specialized manufacturing processes, the cost-effectiveness of our technique at a larger scale remains to be ascertained.

Overall, the MIRA-CRISPR/Cas13a-LFD technique presented in this study provides an efficient, accurate, and user-friendly approach for the rapid detection of *T. vaginalis*, thereby potentially enhancing the diagnosis and management of vaginitis.

## Conclusions

In summary, this study demonstrated that the MIRA-CRISPR/Cas13a-LFD method targeting the repeated DNA element is an efficient, reliable, and rapid detection tool for *T. vaginalis*. The method exhibited superior sensitivity and specificity, with the capacity to discriminate between *T. vaginalis* and other common pathogens in humans. The speed and visual readability of the LFD readout, combined with the high amplification efficiency and selectivity of MIRA–CRISPR/Cas13a, make this method highly suitable for point-of-care testing, particularly in resource-limited settings. Future work will aim to further refine and validate the method in a larger clinical context, potentially integrating it into standard diagnostic procedures to enable quicker and more accurate detection of *T. vaginalis* infections.

### Supplementary Information

**Additional file 1: Figure S1** Primer selection for MIRA of *T. vaginalis* repetitive DNA sequence. (A) Screening of the optimal reverse primer using TV-R1 as the reverse primer. (B) Screening of the optimal forward primer using TV-F3 as the forward primer. (C) Sensitivity testing of the TV-F3/TV-R1 primer pair using positive plasmid template concentrations of (1) 1×10^−2^ ng/μl, (2) 1× 10^−3^ ng/μl, (3) 1× 10^−4^ ng/μl, (4) 1× 10^−5^ ng/μl, (5) 1× 10^−6^ ng/μl, and (6) 1× 10^−7^ ng/μl. NC: negative control.

**Additional file 2: Figure S2** CRISPR probe concentration test.

## Data Availability

All data generated or analyzed during this study are included in this article and additional information files.
