# Supplementary figures and images for: A novel detection method based on MIRA-CRISPR/Cas13a-LFD targeting the repeated DNA sequence of Trichomonas vaginalis
Source: Parasit Vectors. 2024 Jan 8;17:14. doi: 10.1186/s13071-023-06106-3 (PMC10775430; doi:10.1186/s13071-023-06106-3)

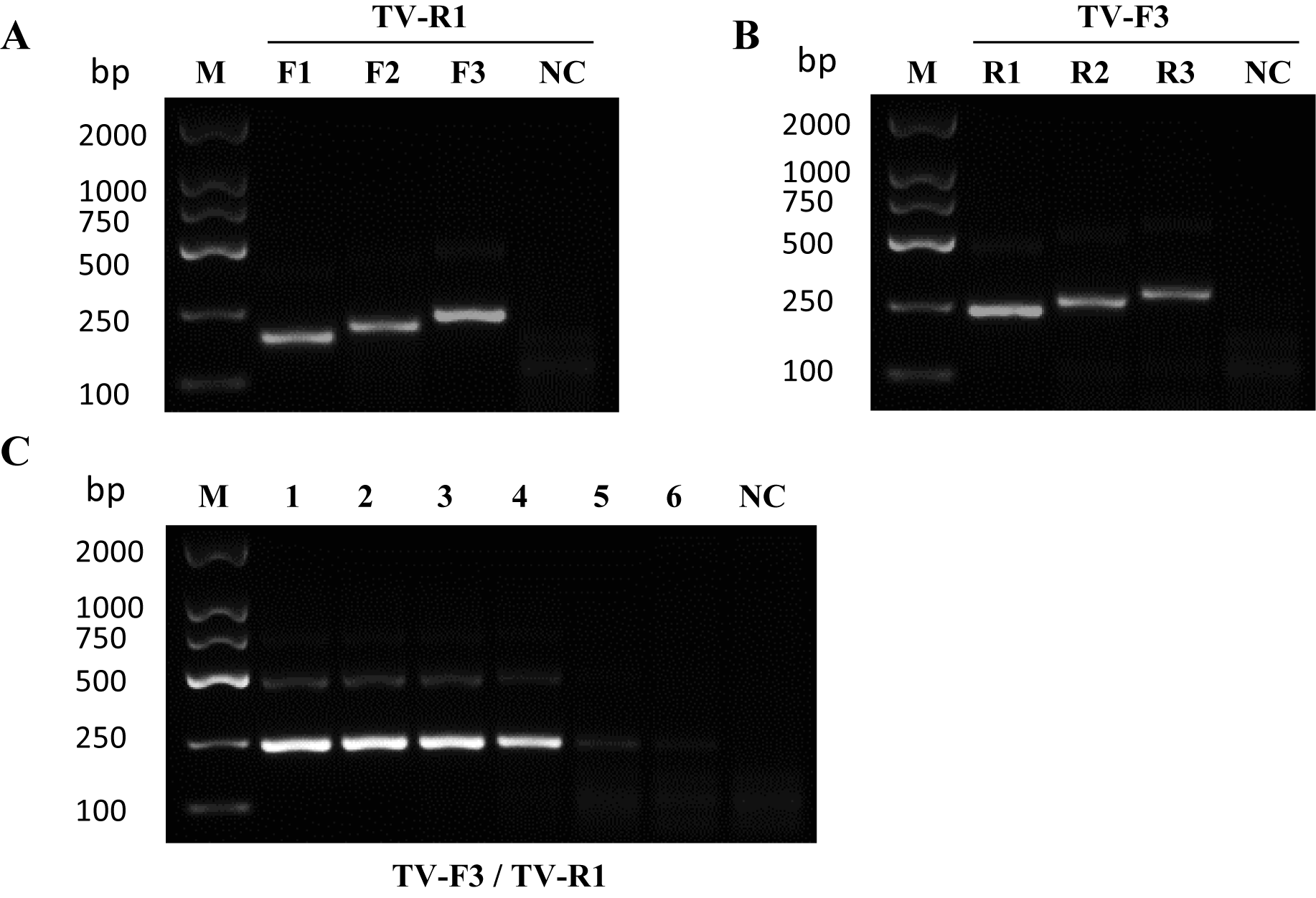

Supplement: Supplementary file 1 — Additional file 1: Figure S1 Primer selection for MIRA of T. vaginalis repetitive DNA sequence. (A) Screening of the optimal reverse primer using TV-R1 as the reverse primer. (B) Screening of the optimal forward primer using TV-F3 as the forward primer. (C) Sensitivity testing of the TV-F3/TV-R1 primer pair using positive plasmid template concentrations of (1) 1×10^−2^ ng/μl, (2) 1× 10^−3^ ng/μl, (3) 1× 10^−4^ ng/μl, (4) 1× 10^−5^ ng/μl, (5) 1× 10^−6^ ng/μl, and (6) 1× 10^−7^ ng/μl. NC: negative control. [file 13071_2023_6106_MOESM1_ESM.tif]

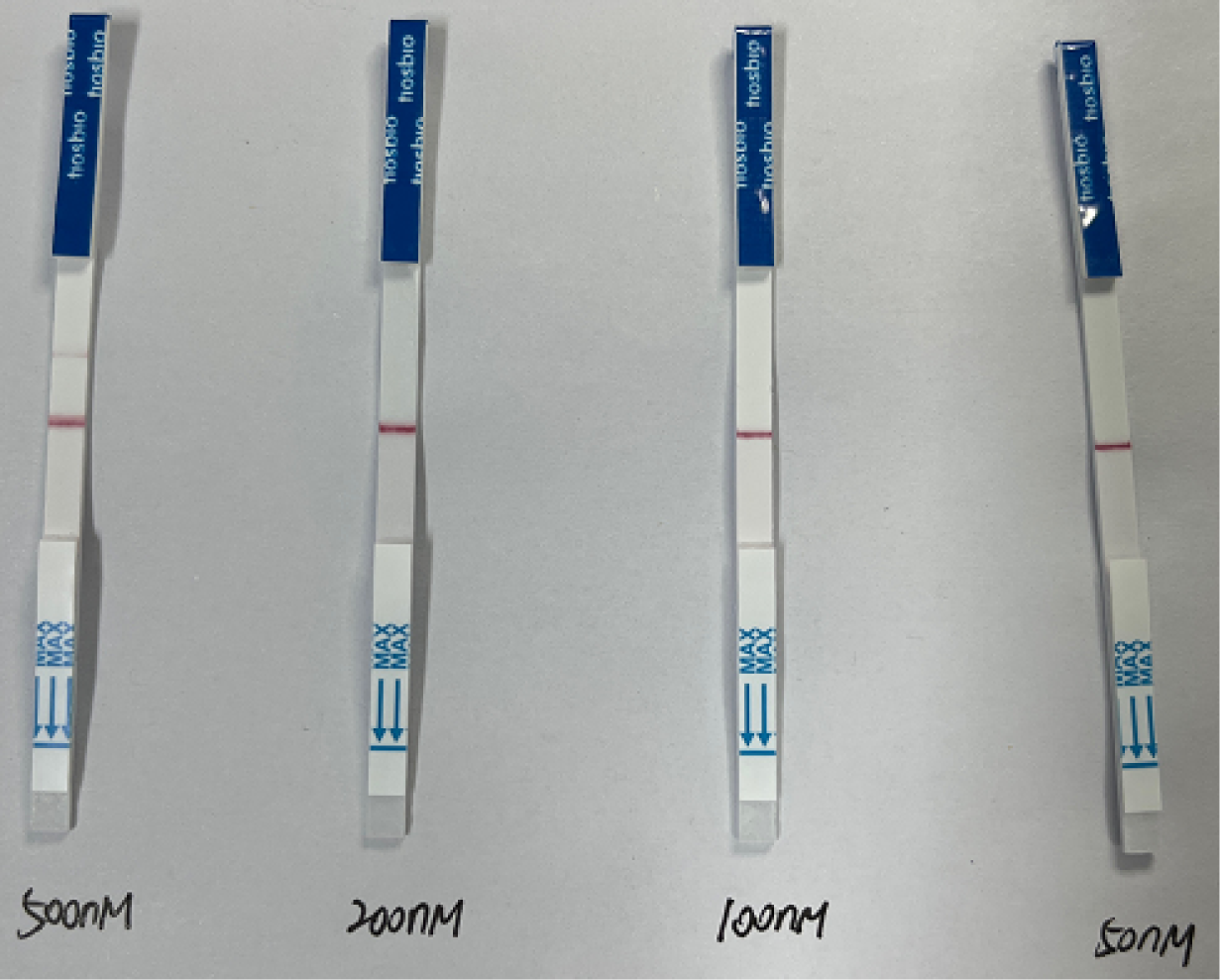

Supplement: Supplementary file 2 — Additional file 2: Figure S2 CRISPR probe concentration test. [file 13071_2023_6106_MOESM2_ESM.tif]
